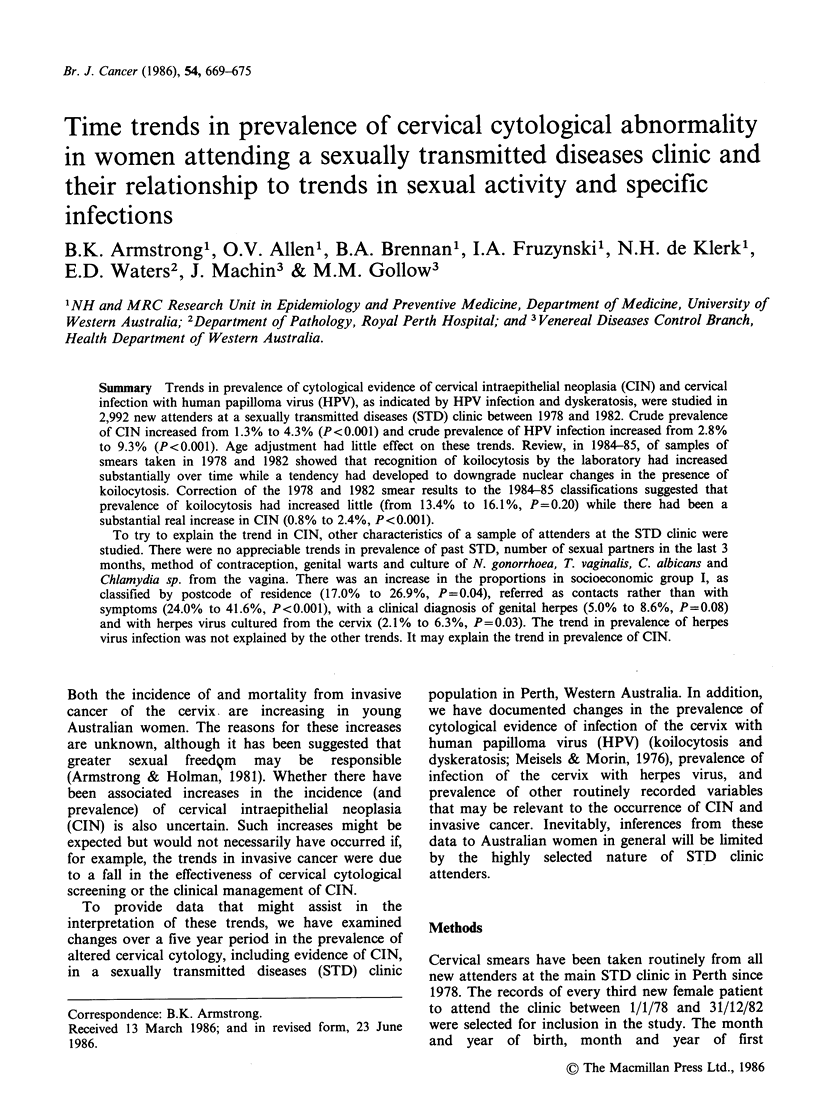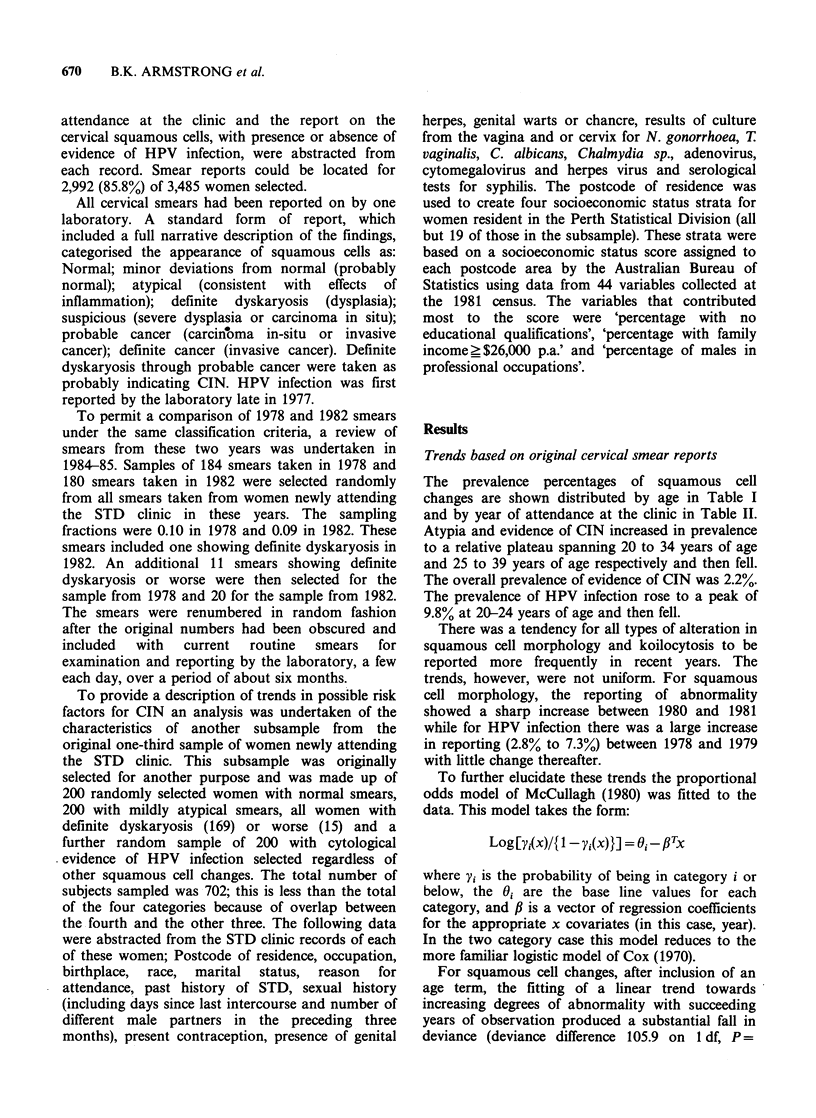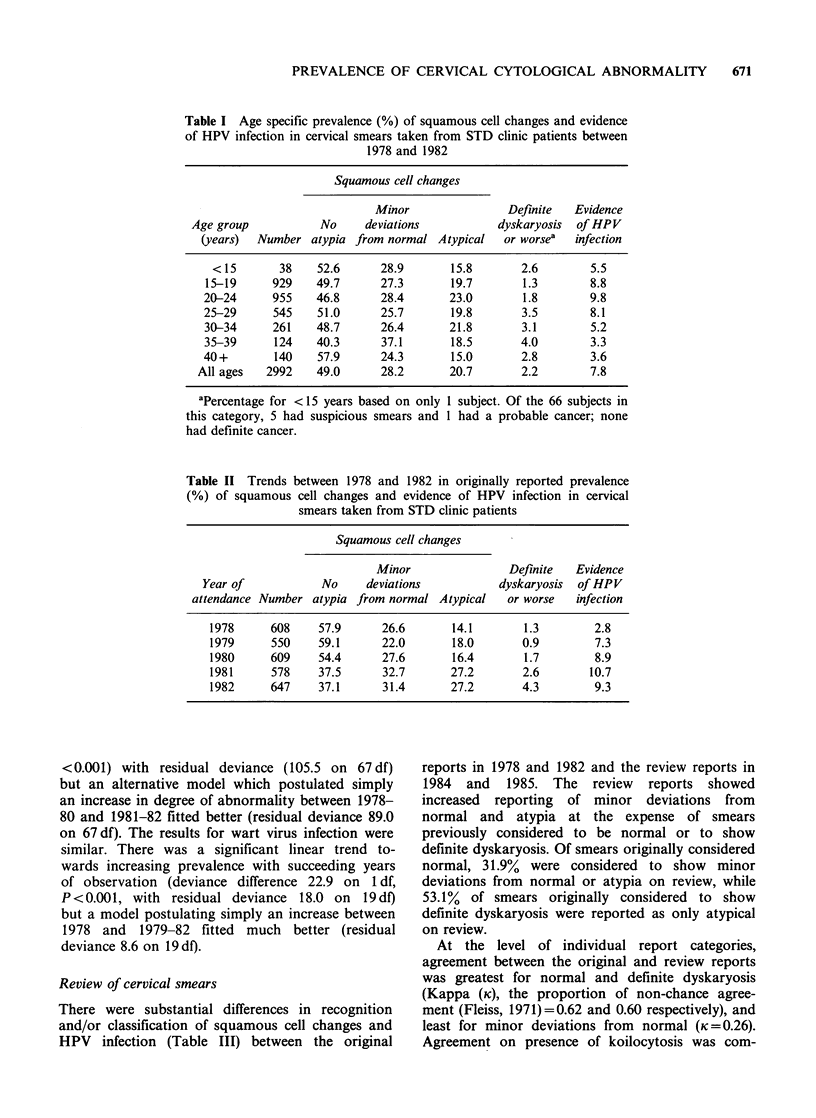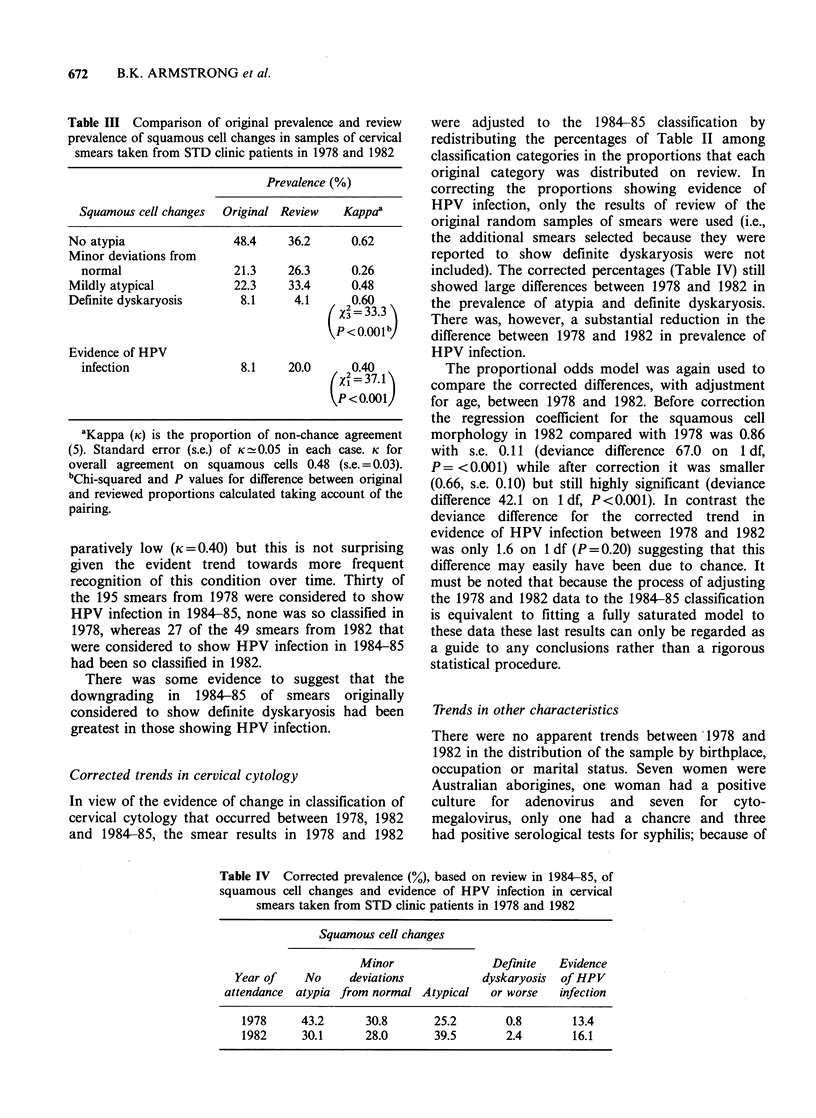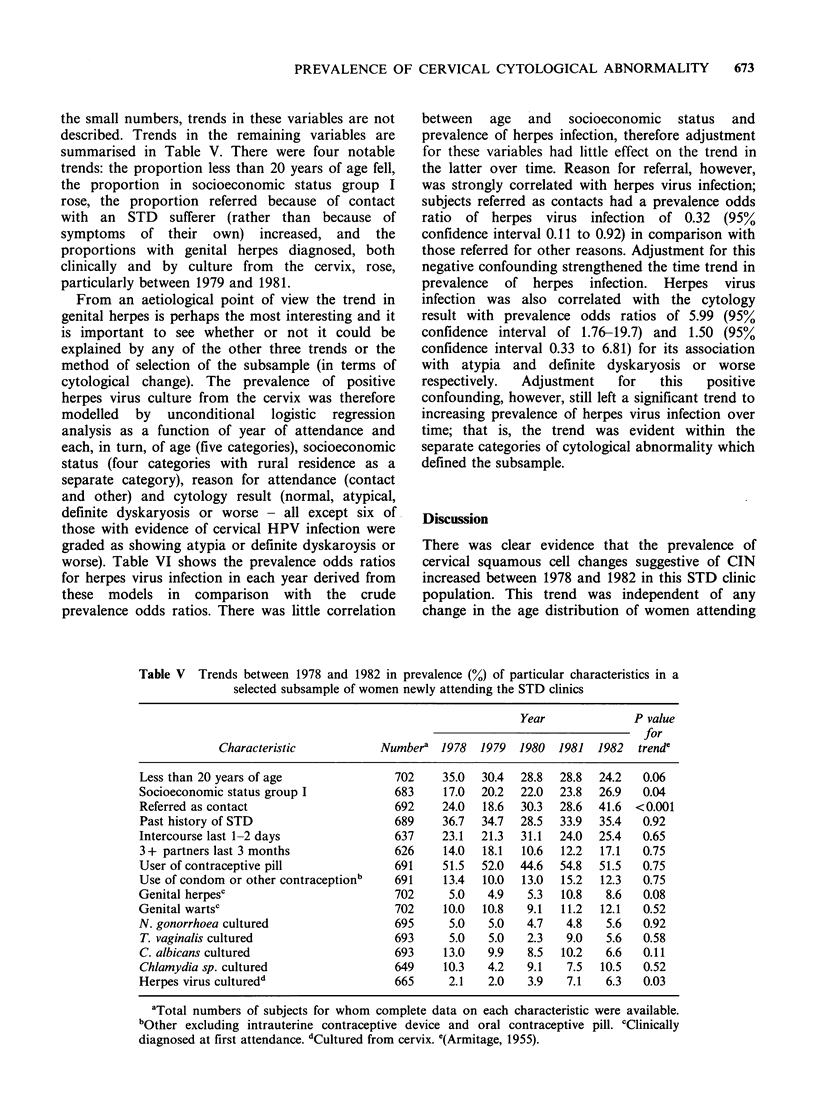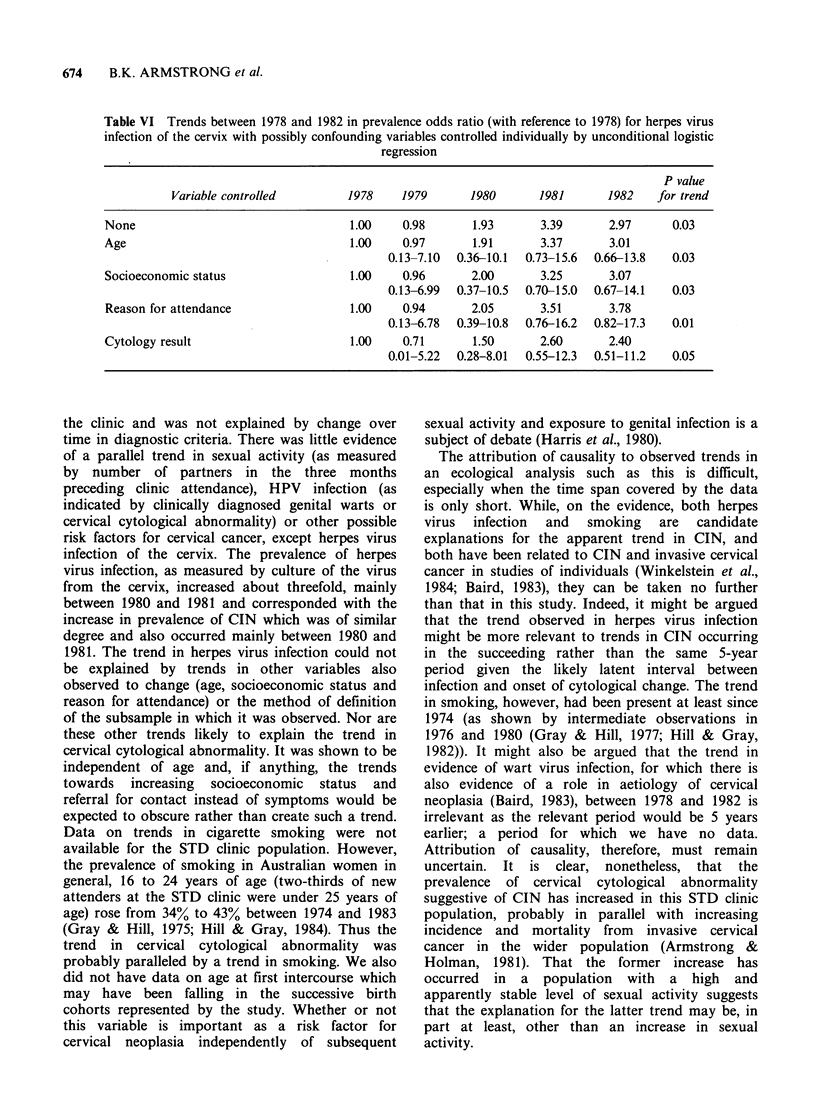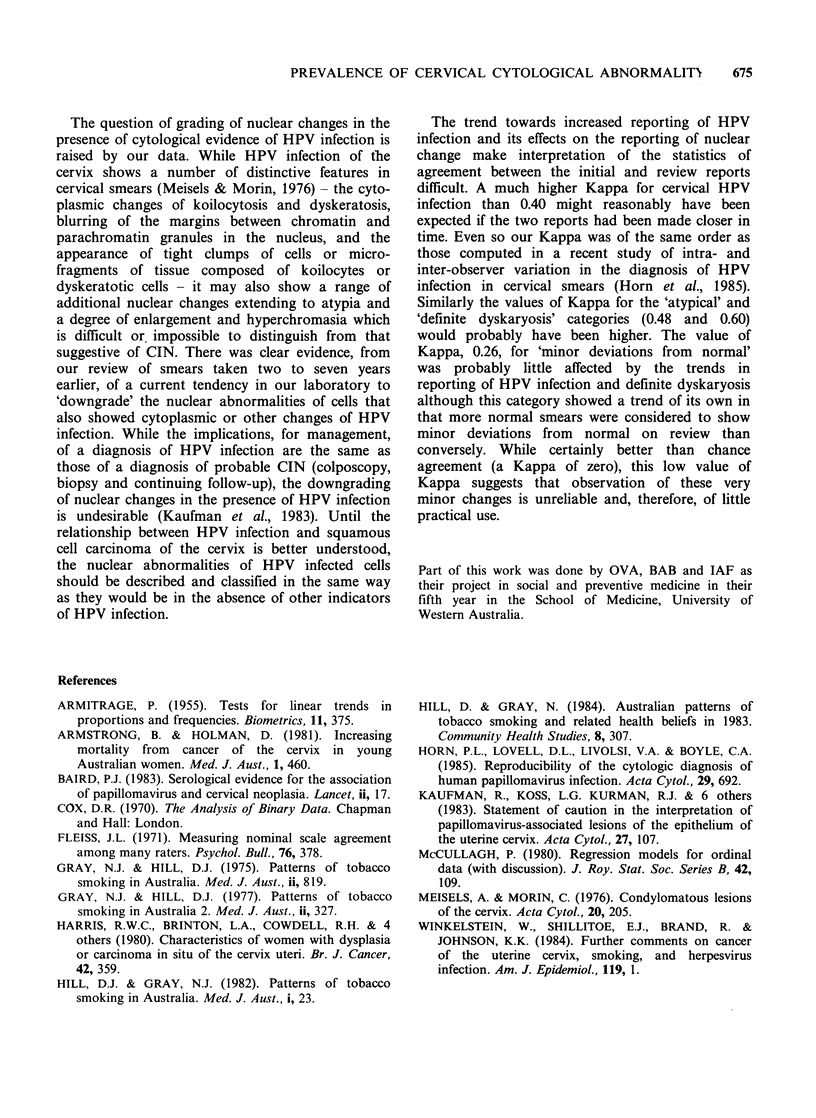# Time trends in prevalence of cervical cytological abnormality in women attending a sexually transmitted diseases clinic and their relationship to trends in sexual activity and specific infections.

**DOI:** 10.1038/bjc.1986.225

**Published:** 1986-10

**Authors:** B. K. Armstrong, O. V. Allen, B. A. Brennan, I. A. Fruzynski, N. H. de Klerk, E. D. Waters, J. Machin, M. M. Gollow

## Abstract

Trends in prevalence of cytological evidence of cervical intraepithelial neoplasia (CIN) and cervical infection with human papilloma virus (HPV), as indicated by HPV infection and dyskeratosis, were studied in 2,992 new attenders at a sexually transmitted diseases (STD) clinic between 1978 and 1982. Crude prevalence of CIN increased from 1.3% to 4.3% (P less than 0.001) and crude prevalence of HPV infection increased from 2.8% to 9.3% (P less than 0.001). Age adjustment had little effect on these trends. Review, in 1984-85, of samples of smears taken in 1978 and 1982 showed that recognition of koilocytosis by the laboratory had increased substantially over time while a tendency had developed to downgrade nuclear changes in the presence of koilocytosis. Correction of the 1978 and 1982 smear results to the 1984-85 classifications suggested that prevalence of koilocytosis had increased little (from 13.4% to 16.1%, P = 0.20) while there had been a substantial real increase in CIN (0.8% to 2.4%, P less than 0.001). To try to explain the trend in CIN, other characteristics of a sample of attenders at the STD clinic were studied. There were no appreciable trends in prevalence of past STD, number of sexual partners in the last 3 months, method of contraception, genital warts and culture of N. gonorrhoea, T. vaginalis, C. albicans and Chlamydia sp. from the vagina. There was an increase in the proportions in socioeconomic group I, as classified by postcode of residence (17.0% to 26.9%, P = 0.04), referred as contacts rather than with symptoms (24.0% to 41.6%, P less than 0.001), with a clinical diagnosis of genital herpes (5.0% to 8.6%, P = 0.08) and with herpes virus cultured from the cervix (2.1% to 6.3%, P = 0.03). The trend in prevalence of herpes virus infection was not explained by the other trends. It may explain the trend in prevalence of CIN.


Br. J. Cancer (1986), 54, 669–675

### Time trends in prevalence of cervical cytological abnormality in women attending a sexually transmitted diseases clinic and their relationship to trends in sexual activity and specific infections

B.K. Armstrong[1], O.V. Allen[1], B.A. Brennan[1], I.A. Fruzynski[1], N.H. de Klerk[1], E.D. Waters[2], J. Machin[3] & M.M. Gollow[3]

[1]*NH and MRC Research Unit in Epidemiology and Preventive Medicine, Department of Medicine, University of Western Australia;* [2]*Department of Pathology, Royal Perth Hospital; and* [3]*Venereal Diseases Control Branch, Health Department of Western Australia.*

**Summary** Trends in prevalence of cytological evidence of cervical intraepithelial neoplasia (CIN) and cervical infection with human papilloma virus (HPV), as indicated by HPV infection and dyskeratosis, were studied in 2,992 new attenders at a sexually transmitted diseases (STD) clinic between 1978 and 1982. Crude prevalence of CIN increased from 1.3% to 4.3% ($P<0.001$) and crude prevalence of HPV infection increased from 2.8% to 9.3% ($P<0.001$). Age adjustment had little effect on these trends. Review, in 1984–85, of samples of smears taken in 1978 and 1982 showed that recognition of koilocytosis by the laboratory had increased substantially over time while a tendency had developed to downgrade nuclear changes in the presence of koilocytosis. Correction of the 1978 and 1982 smear results to the 1984–85 classifications suggested that prevalence of koilocytosis had increased little (from 13.4% to 16.1%, $P=0.20$) while there had been a substantial real increase in CIN (0.8% to 2.4%, $P<0.001$).

To try to explain the trend in CIN, other characteristics of a sample of attenders at the STD clinic were studied. There were no appreciable trends in prevalence of past STD, number of sexual partners in the last 3 months, method of contraception, genital warts and culture of *N. gonorrhoea, T. vaginalis, C. albicans* and *Chlamydia sp.* from the vagina. There was an increase in the proportions in socioeconomic group I, as classified by postcode of residence (17.0% to 26.9%, $P=0.04$), referred as contacts rather than with symptoms (24.0% to 41.6%, $P<0.001$), with a clinical diagnosis of genital herpes (5.0% to 8.6%, $P=0.08$) and with herpes virus cultured from the cervix (2.1% to 6.3%, $P=0.03$). The trend in prevalence of herpes virus infection was not explained by the other trends. It may explain the trend in prevalence of CIN.

Both the incidence of and mortality from invasive cancer of the cervix are increasing in young Australian women. The reasons for these increases are unknown, although it has been suggested that greater sexual freedom may be responsible (Armstrong & Holman, 1981). Whether there have been associated increases in the incidence (and prevalence) of cervical intraepithelial neoplasia (CIN) is also uncertain. Such increases might be expected but would not necessarily have occurred if, for example, the trends in invasive cancer were due to a fall in the effectiveness of cervical cytological screening or the clinical management of CIN.

To provide data that might assist in the interpretation of these trends, we have examined changes over a five year period in the prevalence of altered cervical cytology, including evidence of CIN, in a sexually transmitted diseases (STD) clinic population in Perth, Western Australia. In addition, we have documented changes in the prevalence of cytological evidence of infection of the cervix with human papilloma virus (HPV) (koilocytosis and dyskeratosis; Meisels & Morin, 1976), prevalence of infection of the cervix with herpes virus, and prevalence of other routinely recorded variables that may be relevant to the occurrence of CIN and invasive cancer. Inevitably, inferences from these data to Australian women in general will be limited by the highly selected nature of STD clinic attenders.

### Methods

Cervical smears have been taken routinely from all new attenders at the main STD clinic in Perth since 1978. The records of every third new female patient to attend the clinic between 1/1/78 and 31/12/82 were selected for inclusion in the study. The month and year of birth, month and year of first

Correspondence: B.K. Armstrong.
Received 13 March 1986; and in revised form, 23 June 1986.

© The Macmillan Press Ltd., 1986

attendance at the clinic and the report on the cervical squamous cells, with presence or absence of evidence of HPV infection, were abstracted from each record. Smear reports could be located for 2,992 (85.8%) of 3,485 women selected.

All cervical smears had been reported on by one laboratory. A standard form of report, which included a full narrative description of the findings, categorised the appearance of squamous cells as: Normal; minor deviations from normal (probably normal); atypical (consistent with effects of inflammation); definite dyskaryosis (dysplasia); suspicious (severe dysplasia or carcinoma in situ); probable cancer (carcinoma in-situ or invasive cancer); definite cancer (invasive cancer). Definite dyskaryosis through probable cancer were taken as probably indicating CIN. HPV infection was first reported by the laboratory late in 1977.

To permit a comparison of 1978 and 1982 smears under the same classification criteria, a review of smears from these two years was undertaken in 1984–85. Samples of 184 smears taken in 1978 and 180 smears taken in 1982 were selected randomly from all smears taken from women newly attending the STD clinic in these years. The sampling fractions were 0.10 in 1978 and 0.09 in 1982. These smears included one showing definite dyskaryosis in 1982. An additional 11 smears showing definite dyskaryosis or worse were then selected for the sample from 1978 and 20 for the sample from 1982. The smears were renumbered in random fashion after the original numbers had been obscured and included with current routine smears for examination and reporting by the laboratory, a few each day, over a period of about six months.

To provide a description of trends in possible risk factors for CIN an analysis was undertaken of the characteristics of another subsample from the original one-third sample of women newly attending the STD clinic. This subsample was originally selected for another purpose and was made up of 200 randomly selected women with normal smears, 200 with mildly atypical smears, all women with definite dyskaryosis (169) or worse (15) and a further random sample of 200 with cytological evidence of HPV infection selected regardless of other squamous cell changes. The total number of subjects sampled was 702; this is less than the total of the four categories because of overlap between the fourth and the other three. The following data were abstracted from the STD clinic records of each of these women; Postcode of residence, occupation, birthplace, race, marital status, reason for attendance, past history of STD, sexual history (including days since last intercourse and number of different male partners in the preceding three months), present contraception, presence of genital herpes, genital warts or chancre, results of culture from the vagina and or cervix for *N. gonorrhoea*, *T. vaginalis*, *C. albicans*, *Chalmydia sp.*, adenovirus, cytomegalovirus and herpes virus and serological tests for syphilis. The postcode of residence was used to create four socioeconomic status strata for women resident in the Perth Statistical Division (all but 19 of those in the subsample). These strata were based on a socioeconomic status score assigned to each postcode area by the Australian Bureau of Statistics using data from 44 variables collected at the 1981 census. The variables that contributed most to the score were 'percentage with no educational qualifications', 'percentage with family income ≧ $26,000 p.a.' and 'percentage of males in professional occupations'.

### Results

*Trends based on original cervical smear reports*

The prevalence percentages of squamous cell changes are shown distributed by age in Table I and by year of attendance at the clinic in Table II. Atypia and evidence of CIN increased in prevalence to a relative plateau spanning 20 to 34 years of age and 25 to 39 years of age respectively and then fell. The overall prevalence of evidence of CIN was 2.2%. The prevalence of HPV infection rose to a peak of 9.8% at 20–24 years of age and then fell.

There was a tendency for all types of alteration in squamous cell morphology and koilocytosis to be reported more frequently in recent years. The trends, however, were not uniform. For squamous cell morphology, the reporting of abnormality showed a sharp increase between 1980 and 1981 while for HPV infection there was a large increase in reporting (2.8% to 7.3%) between 1978 and 1979 with little change thereafter.

To further elucidate these trends the proportional odds model of McCullagh (1980) was fitted to the data. This model takes the form:

$$\text{Log}[\gamma_i(x)/\{1-\gamma_i(x)\}] = \theta_i - \beta^T x$$

where $\gamma_i$ is the probability of being in category $i$ or below, the $\theta_i$ are the base line values for each category, and $\beta$ is a vector of regression coefficients for the appropriate $x$ covariates (in this case, year). In the two category case this model reduces to the more familiar logistic model of Cox (1970).

For squamous cell changes, after inclusion of an age term, the fitting of a linear trend towards increasing degrees of abnormality with succeeding years of observation produced a substantial fall in deviance (deviance difference 105.9 on 1 df, $P=$

**Table I** Age specific prevalence (%) of squamous cell changes and evidence of HPV infection in cervical smears taken from STD clinic patients between 1978 and 1982

| | | *Squamous cell changes* | | | | |
|---|---|---|---|---|---|---|
| *Age group (years)* | *Number* | *No atypia* | *Minor deviations from normal* | *Atypical* | *Definite dyskaryosis or worse*[a] | *Evidence of HPV infection* |
| <15 | 38 | 52.6 | 28.9 | 15.8 | 2.6 | 5.5 |
| 15–19 | 929 | 49.7 | 27.3 | 19.7 | 1.3 | 8.8 |
| 20–24 | 955 | 46.8 | 28.4 | 23.0 | 1.8 | 9.8 |
| 25–29 | 545 | 51.0 | 25.7 | 19.8 | 3.5 | 8.1 |
| 30–34 | 261 | 48.7 | 26.4 | 21.8 | 3.1 | 5.2 |
| 35–39 | 124 | 40.3 | 37.1 | 18.5 | 4.0 | 3.3 |
| 40+ | 140 | 57.9 | 24.3 | 15.0 | 2.8 | 3.6 |
| All ages | 2992 | 49.0 | 28.2 | 20.7 | 2.2 | 7.8 |

[a]Percentage for <15 years based on only 1 subject. Of the 66 subjects in this category, 5 had suspicious smears and 1 had a probable cancer; none had definite cancer.

**Table II** Trends between 1978 and 1982 in originally reported prevalence (%) of squamous cell changes and evidence of HPV infection in cervical smears taken from STD clinic patients

| | | *Squamous cell changes* | | | | |
|---|---|---|---|---|---|---|
| *Year of attendance* | *Number* | *No atypia* | *Minor deviations from normal* | *Atypical* | *Definite dyskaryosis or worse* | *Evidence of HPV infection* |
| 1978 | 608 | 57.9 | 26.6 | 14.1 | 1.3 | 2.8 |
| 1979 | 550 | 59.1 | 22.0 | 18.0 | 0.9 | 7.3 |
| 1980 | 609 | 54.4 | 27.6 | 16.4 | 1.7 | 8.9 |
| 1981 | 578 | 37.5 | 32.7 | 27.2 | 2.6 | 10.7 |
| 1982 | 647 | 37.1 | 31.4 | 27.2 | 4.3 | 9.3 |

<0.001) with residual deviance (105.5 on 67 df) but an alternative model which postulated simply an increase in degree of abnormality between 1978–80 and 1981–82 fitted better (residual deviance 89.0 on 67 df). The results for wart virus infection were similar. There was a significant linear trend towards increasing prevalence with succeeding years of observation (deviance difference 22.9 on 1 df, $P<0.001$, with residual deviance 18.0 on 19 df) but a model postulating simply an increase between 1978 and 1979–82 fitted much better (residual deviance 8.6 on 19 df).

### *Review of cervical smears*

There were substantial differences in recognition and/or classification of squamous cell changes and HPV infection (Table III) between the original reports in 1978 and 1982 and the review reports in 1984 and 1985. The review reports showed increased reporting of minor deviations from normal and atypia at the expense of smears previously considered to be normal or to show definite dyskaryosis. Of smears originally considered normal, 31.9% were considered to show minor deviations from normal or atypia on review, while 53.1% of smears originally considered to show definite dyskaryosis were reported as only atypical on review.

At the level of individual report categories, agreement between the original and review reports was greatest for normal and definite dyskaryosis (Kappa ($\kappa$), the proportion of non-chance agreement (Fleiss, 1971) = 0.62 and 0.60 respectively), and least for minor deviations from normal ($\kappa=0.26$). Agreement on presence of koilocytosis was com-

**Table III** Comparison of original prevalence and review prevalence of squamous cell changes in samples of cervical smears taken from STD clinic patients in 1978 and 1982

| | Prevalence (%) | | |
|---|---|---|---|
| *Squamous cell changes* | *Original* | *Review* | *Kappa*[a] |
| No atypia | 48.4 | 36.2 | 0.62 |
| Minor deviations from normal | 21.3 | 26.3 | 0.26 |
| Mildly atypical | 22.3 | 33.4 | 0.48 |
| Definite dyskaryosis | 8.1 | 4.1 | 0.60 ($\chi_3^2=33.3$, $P<0.001$[b]) |
| Evidence of HPV infection | 8.1 | 20.0 | 0.40 ($\chi_1^2=37.1$, $P<0.001$) |

[a]Kappa ($\kappa$) is the proportion of non-chance agreement (5). Standard error (s.e.) of $\kappa \simeq 0.05$ in each case. $\kappa$ for overall agreement on squamous cells 0.48 (s.e. = 0.03). [b]Chi-squared and $P$ values for difference between original and reviewed proportions calculated taking account of the pairing.

paratively low ($\kappa=0.40$) but this is not surprising given the evident trend towards more frequent recognition of this condition over time. Thirty of the 195 smears from 1978 were considered to show HPV infection in 1984–85, none was so classified in 1978, whereas 27 of the 49 smears from 1982 that were considered to show HPV infection in 1984–85 had been so classified in 1982.

There was some evidence to suggest that the downgrading in 1984–85 of smears originally considered to show definite dyskaryosis had been greatest in those showing HPV infection.

### *Corrected trends in cervical cytology*

In view of the evidence of change in classification of cervical cytology that occurred between 1978, 1982 and 1984–85, the smear results in 1978 and 1982 were adjusted to the 1984–85 classification by redistributing the percentages of Table II among classification categories in the proportions that each original category was distributed on review. In correcting the proportions showing evidence of HPV infection, only the results of review of the original random samples of smears were used (i.e., the additional smears selected because they were reported to show definite dyskaryosis were not included). The corrected percentages (Table IV) still showed large differences between 1978 and 1982 in the prevalence of atypia and definite dyskaryosis. There was, however, a substantial reduction in the difference between 1978 and 1982 in prevalence of HPV infection.

The proportional odds model was again used to compare the corrected differences, with adjustment for age, between 1978 and 1982. Before correction the regression coefficient for the squamous cell morphology in 1982 compared with 1978 was 0.86 with s.e. 0.11 (deviance difference 67.0 on 1 df, $P = <0.001$) while after correction it was smaller (0.66, s.e. 0.10) but still highly significant (deviance difference 42.1 on 1 df, $P<0.001$). In contrast the deviance difference for the corrected trend in evidence of HPV infection between 1978 and 1982 was only 1.6 on 1 df ($P=0.20$) suggesting that this difference may easily have been due to chance. It must be noted that because the process of adjusting the 1978 and 1982 data to the 1984–85 classification is equivalent to fitting a fully saturated model to these data these last results can only be regarded as a guide to any conclusions rather than a rigorous statistical procedure.

### *Trends in other characteristics*

There were no apparent trends between 1978 and 1982 in the distribution of the sample by birthplace, occupation or marital status. Seven women were Australian aborigines, one woman had a positive culture for adenovirus and seven for cytomegalovirus, only one had a chancre and three had positive serological tests for syphilis; because of

**Table IV** Corrected prevalence (%), based on review in 1984–85, of squamous cell changes and evidence of HPV infection in cervical smears taken from STD clinic patients in 1978 and 1982

| | Squamous cell changes | | | | |
|---|---|---|---|---|---|
| *Year of attendance* | *No atypia* | *Minor deviations from normal* | *Atypical* | *Definite dyskaryosis or worse* | *Evidence of HPV infection* |
| 1978 | 43.2 | 30.8 | 25.2 | 0.8 | 13.4 |
| 1982 | 30.1 | 28.0 | 39.5 | 2.4 | 16.1 |

the small numbers, trends in these variables are not described. Trends in the remaining variables are summarised in Table V. There were four notable trends: the proportion less than 20 years of age fell, the proportion in socioeconomic status group I rose, the proportion referred because of contact with an STD sufferer (rather than because of symptoms of their own) increased, and the proportions with genital herpes diagnosed, both clinically and by culture from the cervix, rose, particularly between 1979 and 1981.

From an aetiological point of view the trend in genital herpes is perhaps the most interesting and it is important to see whether or not it could be explained by any of the other three trends or the method of selection of the subsample (in terms of cytological change). The prevalence of positive herpes virus culture from the cervix was therefore modelled by unconditional logistic regression analysis as a function of year of attendance and each, in turn, of age (five categories), socioeconomic status (four categories with rural residence as a separate category), reason for attendance (contact and other) and cytology result (normal, atypical, definite dyskaryosis or worse – all except six of those with evidence of cervical HPV infection were graded as showing atypia or definite dyskaroysis or worse). Table VI shows the prevalence odds ratios for herpes virus infection in each year derived from these models in comparison with the crude prevalence odds ratios. There was little correlation between age and socioeconomic status and prevalence of herpes infection, therefore adjustment for these variables had little effect on the trend in the latter over time. Reason for referral, however, was strongly correlated with herpes virus infection; subjects referred as contacts had a prevalence odds ratio of herpes virus infection of 0.32 (95% confidence interval 0.11 to 0.92) in comparison with those referred for other reasons. Adjustment for this negative confounding strengthened the time trend in prevalence of herpes infection. Herpes virus infection was also correlated with the cytology result with prevalence odds ratios of 5.99 (95% confidence interval of 1.76–19.7) and 1.50 (95% confidence interval 0.33 to 6.81) for its association with atypia and definite dyskaryosis or worse respectively. Adjustment for this positive confounding, however, still left a significant trend to increasing prevalence of herpes virus infection over time; that is, the trend was evident within the separate categories of cytological abnormality which defined the subsample.

### Discussion

There was clear evidence that the prevalence of cervical squamous cell changes suggestive of CIN increased between 1978 and 1982 in this STD clinic population. This trend was independent of any change in the age distribution of women attending

**Table V** Trends between 1978 and 1982 in prevalence (%) of particular characteristics in a selected subsample of women newly attending the STD clinics

| *Characteristic* | *Number*[a] | *Year* 1978 | *1979* | *1980* | *1981* | *1982* | *P value for trend*[e] |
|---|---|---|---|---|---|---|---|
| Less than 20 years of age | 702 | 35.0 | 30.4 | 28.8 | 28.8 | 24.2 | 0.06 |
| Socioeconomic status group I | 683 | 17.0 | 20.2 | 22.0 | 23.8 | 26.9 | 0.04 |
| Referred as contact | 692 | 24.0 | 18.6 | 30.3 | 28.6 | 41.6 | <0.001 |
| Past history of STD | 689 | 36.7 | 34.7 | 28.5 | 33.9 | 35.4 | 0.92 |
| Intercourse last 1–2 days | 637 | 23.1 | 21.3 | 31.1 | 24.0 | 25.4 | 0.65 |
| 3+ partners last 3 months | 626 | 14.0 | 18.1 | 10.6 | 12.2 | 17.1 | 0.75 |
| User of contraceptive pill | 691 | 51.5 | 52.0 | 44.6 | 54.8 | 51.5 | 0.75 |
| Use of condom or other contraception[b] | 691 | 13.4 | 10.0 | 13.0 | 15.2 | 12.3 | 0.75 |
| Genital herpes[c] | 702 | 5.0 | 4.9 | 5.3 | 10.8 | 8.6 | 0.08 |
| Genital warts[c] | 702 | 10.0 | 10.8 | 9.1 | 11.2 | 12.1 | 0.52 |
| *N. gonorrhoea* cultured | 695 | 5.0 | 5.0 | 4.7 | 4.8 | 5.6 | 0.92 |
| *T. vaginalis* cultured | 693 | 5.0 | 5.0 | 2.3 | 9.0 | 5.6 | 0.58 |
| *C. albicans* cultured | 693 | 13.0 | 9.9 | 8.5 | 10.2 | 6.6 | 0.11 |
| *Chlamydia sp.* cultured | 649 | 10.3 | 4.2 | 9.1 | 7.5 | 10.5 | 0.52 |
| Herpes virus cultured[d] | 665 | 2.1 | 2.0 | 3.9 | 7.1 | 6.3 | 0.03 |

[a]Total numbers of subjects for whom complete data on each characteristic were available. [b]Other excluding intrauterine contraceptive device and oral contraceptive pill. [c]Clinically diagnosed at first attendance. [d]Cultured from cervix. [e](Armitage, 1955).

**Table VI** Trends between 1978 and 1982 in prevalence odds ratio (with reference to 1978) for herpes virus infection of the cervix with possibly confounding variables controlled individually by unconditional logistic regression

| *Variable controlled* | *1978* | *1979* | *1980* | *1981* | *1982* | *P value for trend* |
|---|---|---|---|---|---|---|
| None | 1.00 | 0.98 | 1.93 | 3.39 | 2.97 | 0.03 |
| Age | 1.00 | 0.97 | 1.91 | 3.37 | 3.01 | |
| | | 0.13–7.10 | 0.36–10.1 | 0.73–15.6 | 0.66–13.8 | 0.03 |
| Socioeconomic status | 1.00 | 0.96 | 2.00 | 3.25 | 3.07 | |
| | | 0.13–6.99 | 0.37–10.5 | 0.70–15.0 | 0.67–14.1 | 0.03 |
| Reason for attendance | 1.00 | 0.94 | 2.05 | 3.51 | 3.78 | |
| | | 0.13–6.78 | 0.39–10.8 | 0.76–16.2 | 0.82–17.3 | 0.01 |
| Cytology result | 1.00 | 0.71 | 1.50 | 2.60 | 2.40 | |
| | | 0.01–5.22 | 0.28–8.01 | 0.55–12.3 | 0.51–11.2 | 0.05 |

the clinic and was not explained by change over time in diagnostic criteria. There was little evidence of a parallel trend in sexual activity (as measured by number of partners in the three months preceding clinic attendance), HPV infection (as indicated by clinically diagnosed genital warts or cervical cytological abnormality) or other possible risk factors for cervical cancer, except herpes virus infection of the cervix. The prevalence of herpes virus infection, as measured by culture of the virus from the cervix, increased about threefold, mainly between 1980 and 1981 and corresponded with the increase in prevalence of CIN which was of similar degree and also occurred mainly between 1980 and 1981. The trend in herpes virus infection could not be explained by trends in other variables also observed to change (age, socioeconomic status and reason for attendance) or the method of definition of the subsample in which it was observed. Nor are these other trends likely to explain the trend in cervical cytological abnormality. It was shown to be independent of age and, if anything, the trends towards increasing socioeconomic status and referral for contact instead of symptoms would be expected to obscure rather than create such a trend. Data on trends in cigarette smoking were not available for the STD clinic population. However, the prevalence of smoking in Australian women in general, 16 to 24 years of age (two-thirds of new attenders at the STD clinic were under 25 years of age) rose from 34% to 43% between 1974 and 1983 (Gray & Hill, 1975; Hill & Gray, 1984). Thus the trend in cervical cytological abnormality was probably paralleled by a trend in smoking. We also did not have data on age at first intercourse which may have been falling in the successive birth cohorts represented by the study. Whether or not this variable is important as a risk factor for cervical neoplasia independently of subsequent sexual activity and exposure to genital infection is a subject of debate (Harris *et al.*, 1980).

The attribution of causality to observed trends in an ecological analysis such as this is difficult, especially when the time span covered by the data is only short. While, on the evidence, both herpes virus infection and smoking are candidate explanations for the apparent trend in CIN, and both have been related to CIN and invasive cervical cancer in studies of individuals (Winkelstein *et al.*, 1984; Baird, 1983), they can be taken no further than that in this study. Indeed, it might be argued that the trend observed in herpes virus infection might be more relevant to trends in CIN occurring in the succeeding rather than the same 5-year period given the likely latent interval between infection and onset of cytological change. The trend in smoking, however, had been present at least since 1974 (as shown by intermediate observations in 1976 and 1980 (Gray & Hill, 1977; Hill & Gray, 1982)). It might also be argued that the trend in evidence of wart virus infection, for which there is also evidence of a role in aetiology of cervical neoplasia (Baird, 1983), between 1978 and 1982 is irrelevant as the relevant period would be 5 years earlier; a period for which we have no data. Attribution of causality, therefore, must remain uncertain. It is clear, nonetheless, that the prevalence of cervical cytological abnormality suggestive of CIN has increased in this STD clinic population, probably in parallel with increasing incidence and mortality from invasive cervical cancer in the wider population (Armstrong & Holman, 1981). That the former increase has occurred in a population with a high and apparently stable level of sexual activity suggests that the explanation for the latter trend may be, in part at least, other than an increase in sexual activity.

The question of grading of nuclear changes in the presence of cytological evidence of HPV infection is raised by our data. While HPV infection of the cervix shows a number of distinctive features in cervical smears (Meisels & Morin, 1976) – the cytoplasmic changes of koilocytosis and dyskeratosis, blurring of the margins between chromatin and parachromatin granules in the nucleus, and the appearance of tight clumps of cells or microfragments of tissue composed of koilocytes or dyskeratotic cells – it may also show a range of additional nuclear changes extending to atypia and a degree of enlargement and hyperchromasia which is difficult or impossible to distinguish from that suggestive of CIN. There was clear evidence, from our review of smears taken two to seven years earlier, of a current tendency in our laboratory to 'downgrade' the nuclear abnormalities of cells that also showed cytoplasmic or other changes of HPV infection. While the implications, for management, of a diagnosis of HPV infection are the same as those of a diagnosis of probable CIN (colposcopy, biopsy and continuing follow-up), the downgrading of nuclear changes in the presence of HPV infection is undesirable (Kaufman *et al.*, 1983). Until the relationship between HPV infection and squamous cell carcinoma of the cervix is better understood, the nuclear abnormalities of HPV infected cells should be described and classified in the same way as they would be in the absence of other indicators of HPV infection.

The trend towards increased reporting of HPV infection and its effects on the reporting of nuclear change make interpretation of the statistics of agreement between the initial and review reports difficult. A much higher Kappa for cervical HPV infection than 0.40 might reasonably have been expected if the two reports had been made closer in time. Even so our Kappa was of the same order as those computed in a recent study of intra- and inter-observer variation in the diagnosis of HPV infection in cervical smears (Horn *et al.*, 1985). Similarly the values of Kappa for the 'atypical' and 'definite dyskaryosis' categories (0.48 and 0.60) would probably have been higher. The value of Kappa, 0.26, for 'minor deviations from normal' was probably little affected by the trends in reporting of HPV infection and definite dyskaryosis although this category showed a trend of its own in that more normal smears were considered to show minor deviations from normal on review than conversely. While certainly better than chance agreement (a Kappa of zero), this low value of Kappa suggests that observation of these very minor changes is unreliable and, therefore, of little practical use.

Part of this work was done by OVA, BAB and IAF as their project in social and preventive medicine in their fifth year in the School of Medicine, University of Western Australia.

**References**

ARMITRAGE, P. (1955). Tests for linear trends in proportions and frequencies. *Biometrics*, **11**, 375.

ARMSTRONG, B. & HOLMAN, D. (1981). Increasing mortality from cancer of the cervix in young Australian women. *Med. J. Aust.*, **1**, 460.

BAIRD, P.J. (1983). Serological evidence for the association of papillomavirus and cervical neoplasia. *Lancet*, **ii**, 17.

COX, D.R. (1970). *The Analysis of Binary Data*. Chapman and Hall: London.

FLEISS, J.L. (1971). Measuring nominal scale agreement among many raters. *Psychol. Bull.*, **76**, 378.

GRAY, N.J. & HILL, D.J. (1975). Patterns of tobacco smoking in Australia. *Med. J. Aust.*, **ii**, 819.

GRAY, N.J. & HILL, D.J. (1977). Patterns of tobacco smoking in Australia 2. *Med. J. Aust.*, **ii**, 327.

HARRIS, R.W.C., BRINTON, L.A., COWDELL, R.H. & 4 others (1980). Characteristics of women with dysplasia or carcinoma in situ of the cervix uteri. *Br. J. Cancer*, **42**, 359.

HILL, D.J. & GRAY, N.J. (1982). Patterns of tobacco smoking in Australia. *Med. J. Aust.*, **i**, 23.

HILL, D. & GRAY, N. (1984). Australian patterns of tobacco smoking and related health beliefs in 1983. *Community Health Studies*, **8**, 307.

HORN, P.L., LOVELL, D.L., LIVOLSI, V.A. & BOYLE, C.A. (1985). Reproducibility of the cytologic diagnosis of human papillomavirus infection. *Acta Cytol.*, **29**, 692.

KAUFMAN, R., KOSS, L.G. KURMAN, R.J. & 6 others (1983). Statement of caution in the interpretation of papillomavirus-associated lesions of the epithelium of the uterine cervix. *Acta Cytol.*, **27**, 107.

McCULLAGH, P. (1980). Regression models for ordinal data (with discussion). *J. Roy. Stat. Soc. Series B*, **42**, 109.

MEISELS, A. & MORIN, C. (1976). Condylomatous lesions of the cervix. *Acta Cytol.*, **20**, 205.

WINKELSTEIN, W., SHILLITOE, E.J., BRAND, R. & JOHNSON, K.K. (1984). Further comments on cancer of the uterine cervix, smoking, and herpesvirus infection. *Am. J. Epidemiol.*, **119**, 1.